# *Mycobacterium tuberculosis* thymidylate synthase gene *thyX* is essential and potentially bifunctional, while *thyA* deletion confers resistance to *p*-aminosalicylic acid

**DOI:** 10.1099/mic.0.053983-0

**Published:** 2012-02

**Authors:** Amanda S. Fivian-Hughes, Joanna Houghton, Elaine O. Davis

**Affiliations:** Division of Mycobacterial Research, MRC National Institute for Medical Research, The Ridgeway, Mill Hill, London NW7 1AA, UK

## Abstract

Thymidylate synthase (TS) enzymes catalyse the biosynthesis of deoxythymidine monophosphate (dTMP or thymidylate), and so are important for DNA replication and repair. Two different types of TS proteins have been described (ThyA and ThyX), which have different enzymic mechanisms and unrelated structures. Mycobacteria are unusual as they encode both *thyA* and *thyX*, and the biological significance of this is not yet understood. *Mycobacterium tuberculosis* ThyX is thought to be essential and a potential drug target. We therefore analysed *M. tuberculosis thyA* and *thyX* expression levels, their essentiality and roles in pathogenesis. We show that both *thyA* and *thyX* are expressed *in vitro*, and that this expression significantly increased within murine macrophages. Under all conditions tested, *thyA* expression exceeded that of *thyX*. Mutational studies show that *M. tuberculosis thyX* is essential, confirming that the enzyme is a plausible drug target. The requirement for *M. tuberculosis thyX* in the presence of *thyA* implies that the essential function of ThyX is something other than dTMP synthase. We successfully deleted *thyA* from the *M. tuberculosis* genome, and this deletion conferred an *in vitro* growth defect that was not observed *in vivo*. Presumably ThyX performs TS activity within *M. tuberculosis* Δ*thyA* at a sufficient rate *in vivo* for normal growth, but the rate *in vitro* is less than optimal. We also demonstrate that *thyA* deletion confers *M. tuberculosis* *p*-aminosalicylic acid resistance, and show by complementation studies that ThyA T202A and V261G appear to be functional and non-functional, respectively.

## Introduction

Thymidylate synthase (TS) enzymes catalyse the reductive methylation of dUMP to synthesize dTMP or thymidylate, and so are important for DNA replication and repair. Two different types of TS proteins have been described, which have different enzymic mechanisms and unrelated structures, and are therefore thought to have evolved independently (Carreras & Santi, 1995; Koehn *et al.*, 2009; Kuhn *et al.*, 2002; Myllykallio *et al.*, 2002). ThyA (encoded by *thyA*), known as the classical or conventional TS, uses methylene tetrahydrofolate (mTHF) as the methyl donor and reductant, yielding dihydrofolate (DHF) as a by-product (Carreras & Santi, 1995). As reduced folate derivatives are essential for a variety of biological processes, DHF is converted back to tetrahydrofolate (THF) by dihydrofolate reductase (DHFR; encoded by *folA* in *Escherichia coli* and *dfrA* in *Mycobacterium tuberculosis*). The more recently discovered ThyX (encoded by *thyX*), known as the alternative or flavin-dependent TS, also uses mTHF as the methyl donor, yielding THF, but uses reduced flavin adenine dinucleotide (FADH_2_) as the reductant (Gattis & Palfey, 2005; Kuhn *et al.*, 2002; Myllykallio *et al.*, 2002). In both cases THF is converted back to mTHF by serine hydroxymethyltransferase.

Most organisms contain either *thyA* or *thyX*, and a TS-encoding gene is usually essential (or its deletion creates a thymidine auxotroph if the organism also encodes thymidine kinase). ThyA and ThyX clearly perform the same function, as complementation experiments show that one can functionally replace the other. For example, the thymidine auxotrophy of *E. coli* lacking *thyA* can be complemented by *thyX* from various organisms, including *Helicobacter pylori* (Myllykallio *et al.*, 2002) and *Campylobacter jejuni* (Giladi *et al.*, 2002). Also, *Halobacterium volcanii thyA* can be functionally replaced by *Halobacterium salinarum thyX* (Giladi *et al.*, 2002). When *thyX* was deleted from *Rhodobacter capsulatus*, both *thyA* and *folA* from *Rhodobacter sphaeroides* were required for full complementation, although *thyA* alone did partially complement the defect (Leduc *et al.*, 2007). This result is not surprising, as the chemical reaction performed by ThyA requires high DHFR activity (Giladi *et al.*, 2002), and most ThyX-dependent bacteria (including *R. capsulatus*) do not contain a *folA* homologue (Leduc *et al.*, 2007; Myllykallio *et al.*, 2002, 2003).

Corynebacteria (including mycobacteria) are unusual as they encode both *thyA* and *thyX* (and a *folA* homologue; Myllykallio *et al.*, 2002), and the biological significance of this is not yet understood. It is known that both *thyA* and *thyX* from *M. tuberculosis* (Rengarajan *et al.*, 2004; Sampathkumar *et al.*, 2005) and *Corynebacterium glutamicum* (Park *et al.*, 2010) can functionally complement an *E. coli thyA* deletion strain. ThyA and ThyX from these two species exhibit 72 and 63 % sequence similarity at the amino acid level, respectively (Kan *et al.*, 2010; Park *et al.*, 2010). Both *M. tuberculosis* ThyA and ThyX proteins have been shown to have TS activity *in vitro*, with ThyA being substantially more efficient than ThyX (Hunter *et al.*, 2008). However, purified *C. glutamicum* ThyX does not exhibit any detectable TS activity (Kan *et al.*, 2010). Mutational studies of *C. glutamicum* show that *thyX* is not essential for *in vitro* growth, but is thought to play a role in survival during stationary phase (Park *et al.*, 2010). *M. tuberculosis thyA* is not thought to be essential, as *thyA* transposon mutants are viable (Sassetti *et al.*, 2003), and some clinical *M. tuberculosis p*-aminosalicylic acid (PAS)-resistant strains contain point mutations within *thyA* (Leung *et al.*, 2010; Mathys *et al.*, 2009; Rengarajan *et al.*, 2004; Zhang *et al.*, 2007). Unlike *thyX* of *C. glutamicum*, *M. tuberculosis thyX* is thought to be essential (Sassetti *et al.*, 2003), and there is pharmacological interest in this enzyme (and ThyX from other pathogenic organisms), as *thyX* is absent from the human genome (Myllykallio *et al.*, 2002, 2003). Indeed, the structural, functional and kinetic analysis of *M. tuberculosis* ThyX is already under way so that the rational design of ThyX inhibitors can begin (Hunter *et al.*, 2008; Sampathkumar *et al.*, 2005, 2006; Ulmer *et al.*, 2008). This is of importance, as *M. tuberculosis* kills approximately two million people annually (WHO, 2009), and no novel antitubercular compound has been introduced since rifampicin in 1963 (Ahmed & Hasnain, 2004). The increasing incidence of infections with multidrug-resistant (MDR) and extensively drug-resistant (XDR) *M. tuberculosis* strains, and the associated mortality rates and high cost of second-line antibiotics, heighten the urgent demand for compounds with novel modes of action to treat tuberculosis (Haydel, 2010; Koul *et al.*, 2011; Zignol *et al.*, 2006).

The main aim of this study was to investigate the biological roles of *M. tuberculosis thyA* and *thyX* by studying their expression levels, essentiality and roles in pathogenesis, to establish whether either or both of the enzymes that they encode are plausible drug targets.

## Methods

### 

#### Bacterial strains and culture conditions.

*E. coli* strain DH5α (Invitrogen) was used for all plasmid construction, and strain XL1-Blue (Stratagene) was used for site-directed mutagenesis (SDM). *E. coli* was grown at 37 °C on Luria–Bertani (LB) agar, or in LB broth with shaking at 250 r.p.m. Where appropriate, *E. coli* culture media was supplemented with 100 µg ampicillin ml^−1^, 50 µg kanamycin ml^−1^, 20 µg gentamicin ml^−1^ and/or 200 µg X-Gal ml^−1^.

*M. tuberculosis* H37Rv was used as wild-type and as the parental strain for deletion strains. *M. tuberculosis* was usually grown at 37 °C on Middlebrook 7H11 agar (Difco) or in modified Dubos medium (Difco), both supplemented with 4 % Dubos Medium Albumin (Difco) and 0.5 or 0.2 % (w/v) glycerol, respectively. However, 7H11 agar was supplemented with 10 % Middlebrook oleic acid-albumin-dextrose-catalase (OADC) enrichment (Difco) and 0.5 % (w/v) glycerol when enumerating mycobacterial c.f.u., and the concentration of albumin was increased to 10 % for Δ*thyA* liquid cultures due to bacterial clumping. *M. tuberculosis* liquid cultures were grown in a roller incubator at 2 r.p.m. or in an SB3 Tube Rotator (Stewart) at 30 r.p.m. Where appropriate, *M. tuberculosis* culture medium was supplemented with 25 µg kanamycin ml^−1^, 15 µg gentamicin ml^−1^, 50 µg X-Gal ml^−1^ and/or 20 mg sucrose ml^−1^.

#### Stress exposure.

*M. tuberculosis* H37Rv was grown with rolling at 2 r.p.m. to OD_600_ ~0.4, and then 25 ml aliquots were placed into six 50 ml Falcon tubes. For oxidative stress, t-butyl hydroperoxide (TBHP) was added to three cultures to a final concentration of 100 µM, and the other three cultures were untreated controls. For starvation stress, bacteria were harvested by centrifugation, and three cultures were resuspended in 1×PBS (pH 7.4), while the three control cultures were resuspended in Dubos medium. For nitrosative stress, bacteria were harvested and resuspended in acidified Dubos medium (pH 5.4), and sodium nitrite was added to three cultures to a final concentration of 2 mM; the remaining three acidified cultures were controls for nitrosative stress, but also acted as acid-stress cultures compared with the aforementioned control cultures resuspended in non-acidified Dubos medium. Falcon tube cultures were then incubated for 24 h with rotation at 30 r.p.m., and RNA was subsequently prepared.

#### Murine bone marrow-derived macrophages (BMDM) infection model.

Monocytes were isolated from the hind legs of female 6–8 week old BALB/c mice, allowed to differentiate into macrophages for 6 days and flushed from Petri dishes as described elsewhere (Spivey *et al.*, 2011). Macrophages were seeded into either tissue culture treated 75 cm^2^ flasks (for bacterial RNA isolation) or 24-well plates (for bacterial survival assays) at a density of 2.6×10^6^ cells per flask or 2×10^5^ cells per well. Where appropriate, murine interferon-γ (IFN-γ; Roche) was added at a concentration of 10 ng ml^−1^. Flasks/plates were incubated at 37 °C with 5 % CO_2_ overnight to allow adherence and activation. *M. tuberculosis* cultures were grown to OD_600_ ~0.5, and washed once with, and then resuspended in, 1× PBS containing 0.05 % Tween 80 (PBS-T). The cells were centrifuged at 300 r.p.m. to remove any aggregated cells, and an appropriate dilution in PBS-T was prepared. Macrophages were infected at an m.o.i. of either 5 : 1 in flasks or 0.1 : 1 in wells for 24 or 4.5 h, respectively. After this time the infected macrophages were washed three times with warm HBSS (Invitrogen), and fresh medium, with or without 10 ng IFN-γ ml^−1^, was added as appropriate. Flasks/plates were incubated at 37 °C with 5 % CO_2_. Mycobacterial RNA was prepared from macrophages in flasks after a further 24 h incubation, i.e. 48 h post-infection (see next section). The survival and multiplication of *M. tuberculosis* were determined by enumerating c.f.u. within macrophages in wells at various time points.

#### RNA purification and quantitative real-time RT-PCR (qRT-PCR) analysis.

To isolate mycobacterial RNA from infected BMDM, medium was removed from the flasks and replaced with two volumes (26 ml) of guanidine thiocyanate (GTC) solution (4 M GTC, 25 mM sodium citrate, 0.5 % *N*-laurylsarcosine sodium salt, 0.5 % Tween 80 and 100 mM β-mercaptoethanol). Cells were scraped from each flask with a cell scraper, the solution was poured into a 50 ml Falcon tube, vortexed vigorously and centrifuged, and RNA was then prepared from the pellet using a FastRNA Pro Blue kit (Qbiogene).

Additional RNA purification, removal of contaminating DNA, conversion to cDNA and qRT-PCR were performed as described previously (Fivian-Hughes & Davis, 2010), except that data were normalized to *rrs* (16S rRNA).

#### Plasmid construction.

The plasmids used in this study are listed and their construction described in Table 1. Primers are listed in Table 2. SDM was performed using the QuikChange SDM kit (Stratagene). All plasmids were verified by DNA sequencing.

**Table 1.** Plasmids used in this study Kanamycin^R^ and ampicillin^R^, kanamycin and ampicillin resistance, respectively.

Name	Description and construction	Reference or source
pBackbone	Mycobacterial suicide vector (kanamycin^R^ and ampicillin^R^)	Gopaul (2002)
pGoal17	Plasmid containing the *sacB*/*lacZ* cassette (ampicillin^R^)	Parish & Stoker (2000)
pKP186	Integrating mycobacterial cloning vector that does not contain integrase (kanamycin^R^)	Rickman *et al.* (2005)
pKP203	pKP186 derivative with a gentamicin-resistance cassette replacing the kanamycin-resistance cassette	Rossi *et al.* (2005)
pBS-Int	Mycobacterial suicide vector containing integrase (ampicillin^R^), electroporated in conjunction with pKP186 and its derivatives	Springer *et al.* (2001)
pJH-*thyX*	*thyX* deletion plasmid; pBackbone containing *thyX* 5′ and 3′ flanking regions, and the *sacB*/*lacZ* cassette*	This study
pJH-*thyA*	*thyA* deletion plasmid; pBackbone containing *thyA* 5′ and 3′ flanking regions, and the *sacB*/*lacZ* cassette*	This study
pASF59	*thyX* complementation plasmid; pKP203 containing *thyX*, including 523 bp upstream and 53 bp downstream of the annotated gene. The 1375 bp PCR product from primers *thyX*compF and *thyX*compR was inserted into *Pvu*II	This study
pASF63	*thyA* complementation plasmid; pKP186 containing *thyA*, including 340 bp upstream and 110 bp downstream of the annotated gene. The 1249 bp PCR product from primers *thyA*compF and *thyA*compR was cloned into *Bam*HI and *Not*I	This study
pASF70	pASF63 derivative containing the T202A (ACC→GCC) mutation in *thyA*; SDM primers T202A F and T202A R were used	This study
pASF71	pASF63 derivative containing the V261G (GTC→GGC**)** mutation in *thyA*; SDM primers V261G F and V261G R were used	This study

* Construction of deletion plasmids involved multiple cloning steps (refer to Methods).

**Table 2.** Primers and oligonucleotides used in this study

Name	Sequence (5′–3′)
16srRNAF*	AAGAAGCACCGGCCAACTAC
16srRNAR*	TCGCTCCTCAGCGTCAGTTA
*thyA* qF*	TGCACGAGCACGGAGTCA
*thyA* qR*	CCCGAGTTCGCCTGTATCAC
*thyX* qF*	GCTACCTCCGGCACATCATC
*thyX* qR*	GACACGCTGGCATGCTCTAG
*dfrA* qF*	CCGGCGAAATGTCGTACTG
*dfrA* qR*	CTCCTCGAGTGAACCGACAAC
*thyX* 5′ For	GACTGCGGCCGCAACGAGGAAGAC
*thyX* 5′ Rev	GGTCTAGACCACGGCGCTCACCTTAG
*thyX* 3′ For	GTGGCGACCAGCCCGTTCTAGACCGAAG
*thyX* 3′ Rev	GGCCACCCTTTGGGGATCCTTTAGA
*thyX*compF	GGGAGCAACTGCGGCCGCTTAGATG
*thyX*compR	GACGGTGGTCACGGATCCCAAGGTTAC
*thyX* CheckF	CGGCCGAAGGAATGGAGGAGT
*thyX* CheckR	GGCGACGGCGGTGAAATG
*thyA* 5′ For	GTGGAAAGTGAGCTCACGCACTGC
*thyA* 5′ Rev	GTTTCTAGAACGAAGCGCAGCAGGTCC
*thyA* 3′ For	GATCAAAGCTCTAGACGCGGTAT
*thyA* 3′ Rev	AAGTCGAGATGGATCCCTAGCCTTACCT
*thyA* probeF	CGCGCCGACGACAATGAA
*thyA* probeR	GCAGCGGCCGGACTTTAGC
*thyA*compF	GCGGTAGTACACGTCGGGATCCAGAAC
*thyA*compR	CCGCCGCGGCCGTGACAC
T202A F	GGTCGGCGAGTTCATCTGGGCCGGTGGCGACTGCCACATC
T202A R	GATGTGGCAGTCGCCACCGGCCCAGATGAACTCGCCGACC
V261G F	CCGGCGATCAAAGCTCCAGGCGCGGTATGAGGCGCGCCG
V261G R	CGGCGCGCCTCATACCGCGCCTGGAGCTTTGATCGCCGG

* qRT-PCR primers.

#### Creating *M. tuberculosis* deletion strains.

The 5′ and 3′ flanking regions of interest were amplified by PCR and were sequentially cloned into the suicide plasmid pBackbone (Gopaul, 2002), using restriction enzymes for which sites were incorporated into the primers. The 6.4 kb *sacB/lacZ* cassette from pGoal17 (Parish & Stoker, 2000) was cloned into the unique *Pac*I site of the plasmids. Deletion plasmids were electroporated into *M. tuberculosis* H37Rv, and screening and counter-selection processes were performed as outlined elsewhere (Parish & Stoker, 2000). Primers were designed such that 750 bp of the 792 bp *thyA* gene was deleted, leaving only the first 32 bp and the last 10 bp, separated by an *Xba*I site. Likewise, 738 bp of the 753 bp *thyX* gene was deleted, leaving only the first 4 bp and the last 11 bp, again separated by an *Xba*I site.

#### Southern blotting.

Putative *M. tuberculosis thyA* deletion strains were confirmed by Southern blot analysis performed as described previously (Fivian-Hughes & Davis, 2010), except that genomic DNA was digested with *Pst*I, and the probe consisted of a 240 bp PCR product generated from primers *thyA* probeF and *thyA* probeR.

#### Assessing *in vitro* growth.

*M. tuberculosis* strains were grown with rolling at 2 r.p.m. to OD_600_ ~0.5. These cultures were used to inoculate fresh media to OD_600_ ~0.02, and the OD_600_ was then monitored for 14 days.

#### Mouse infection studies.

*M. tuberculosis* strains were prepared as for macrophage infections, except that the final resuspension in PBS did not contain Tween 80. Female 6–8 week old BALB/c mice were injected intravenously with approximately 1×10^6^ bacteria. The survival and multiplication of *M. tuberculosis* were determined by enumerating c.f.u. within the lungs and spleens of four mice at each time point.

#### Assessing PAS susceptibility.

*M. tuberculosis* strains were grown with rotation at 30 r.p.m. to OD_600_ ~0.4. Cultures were serially diluted in PBS-T and were plated onto agar containing 0, 0.1, 1, 10 or 100 µg PAS ml^−1^. c.f.u. were enumerated and percentage survival relative to untreated samples was calculated.

## Results

### *thyA* is expressed at a higher level than *thyX*

**Fig. 1.**
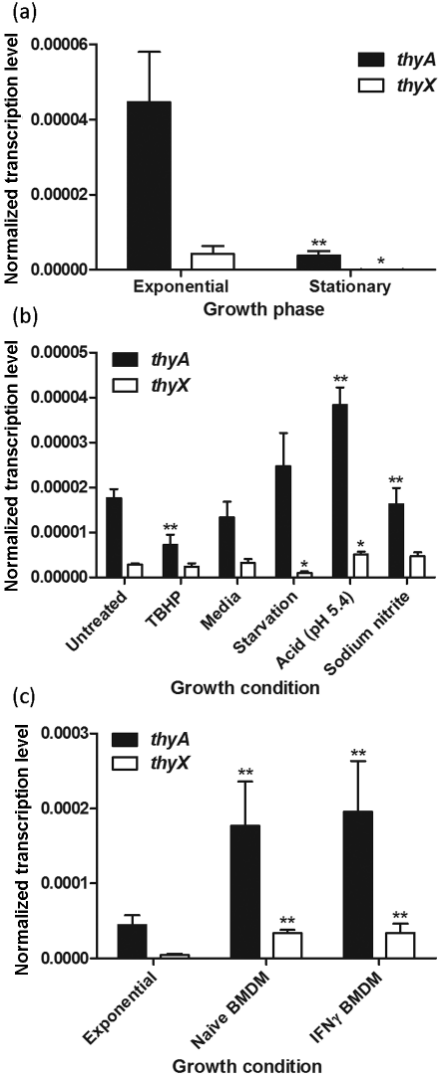
*In vitro* and *in vivo* expression levels of *thyA* and *thyX.* qRT-PCR was performed on RNA extracts of *M. tuberculosis* H37Rv (a) grown to exponential and stationary phases (OD_600_ 0.8 and 4.0, respectively), (b) after 24 h exposure to different stress conditions and (c) after 48 h intracellular growth within murine BMDM. The data are means of at least three biological replicates or at least six infections (each assayed in triplicate), normalized to corresponding *rrs* (16S rRNA) values; error bars, sd. Oxidative stress was modelled using 100 µM TBHP, and untreated cultures acted as controls. Starvation and acid stress were modelled by resuspending cells in PBS or acidified media (pH 5.4), respectively, and cells resuspended in media (Media) acted as control cultures. Nitrosative stress was modelled by resuspending cells in acidified media containing 2 mM sodium nitrite, and cells resuspended in acidified media acted as control cultures. Significant differences in *thyA* or *thyX* expression from equivalent exponential phase or appropriate control levels are indicated (* or **; Student’s *t* test, *P*<0.05 or *P*<0.01, respectively).

Earlier microarray analysis suggested that *M. tuberculosis thyX* mRNA was undetectable during both *in vitro* and *in vivo* growth (Talaat *et al.*, 2004). Therefore, we first decided to study *in vitro* expression levels of *thyA* and *thyX* using qRT-PCR, which is a more sensitive technique than microarray technology. Both *thyA* and *thyX* were detectable by qRT-PCR from *M. tuberculosis* H37Rv grown to exponential phase (Fig. 1a). The expression of both of these genes was significantly reduced upon reaching stationary phase (Fig. 1a), as one might expect when the DNA replication rate is lower, to the point that *thyX* was undetectable. Interestingly, *thyA* expression was significantly higher than *thyX* expression in both growth phases (Fig. 1a; Student’s *t* tests, *P*<0.05 and *P*<0.01 for exponential and stationary phase, respectively), yet *thyX* is the gene predicted to be essential (Sassetti *et al.*, 2003).

It has been postulated that mycobacteria might preferentially use ThyA or ThyX under different growth conditions (Sampathkumar *et al.*, 2005). We therefore assessed *thyA* and *thyX* expression after 24 h exposure to various *in vitro* stresses, in search of a condition where *thyX* expression might be increased. Expression of *thyA* was significantly decreased by 2.4-fold upon both oxidative and nitrosative stress, and was significantly increased by 2.9-fold upon acid stress, when compared with appropriate controls (Fig. 1b). Expression of *thyX* was significantly decreased by 3.2-fold upon starvation, and was significantly increased by 1.6-fold upon acid stress (Fig. 1b). For all of the *in vitro* growth conditions tested, *thyA* expression remained significantly higher than that of *thyX* (Fig. 1b; Student’s *t* tests, *P*<0.01 for all test conditions except TBHP, where *P*<0.05).

It should be noted that the exponential phase cultures in Fig. 1(a) are essentially the same as the untreated cultures in Fig. 1(b), but *thyA* expression is significantly lower in Fig. 1(b) (Student’s *t* test, *P* = 0.01). This may be due to the level of culture aeration, as 50 ml cultures were grown in 1 l bottles with rolling at 2 r.p.m. for the experiment shown in Fig. 1(a), while the data shown in Fig. 1(b) were obtained from 25 ml cultures incubated in 50 ml Falcon tubes rotating at 30 r.p.m. (see Methods). It is not known why the level of aeration would have this effect on *thyA* expression.

### *thyA* and *thyX* expression significantly increases during macrophage infection

To investigate whether *thyA* or *thyX* expression is altered *in vivo*, *M. tuberculosis* H37Rv was used to infect murine BMDM at an m.o.i. of 5 : 1. Mycobacterial RNA was isolated 48 h post-infection using a solution containing GTC (see Methods). GTC solution lyses the macrophages and stabilizes the mycobacterial RNA, thus allowing the separation of bacteria from the macrophage lysate without compromising the mycobacterial RNA (Butcher *et al.*, 1998). No *thyA* or *thyX* expression was detected during preliminary qRT-PCR experiments using uninfected macrophages (data not shown), indicating either that host RNA was absent or that any contaminating host RNA could not be amplified with the mycobacterial-specific primers. Expression of *thyA* and *thyX* was significantly increased within both naive and IFN-γ-activated macrophages, when compared with *in vitro* exponential expression levels (by 4.0-fold and 4.4-fold for *thyA*, and 7.9-fold and 8.0-fold for *thyX*, respectively; Fig. 1c). However, *thyA* expression remained significantly higher than *thyX* expression within both naive and activated macrophages (Fig. 1c; Student’s *t* tests, *P*<0.01).

### *thyX* (Rv2754c) is essential

**Fig. 2.**
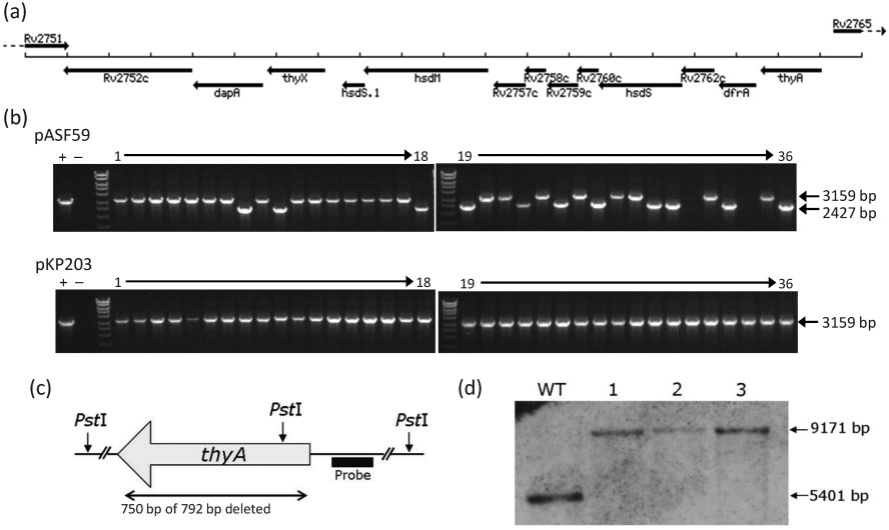
Deletion of *thyX* and *thyA* from the *M. tuberculosis* genome. (a) Schematic diagram showing the relative locations of *thyX* and *thyA* within the *M. tuberculosis* H37Rv genome (adapted from TubercuList; http://genolist.pasteur.fr/TubercuList/). (b) PCR analysis for the *thyX* deletion within 36 colonies selected from the *thyX* single cross-over strain either with (pASF59) or without (pKP203) an additional copy of *thyX* present. The primers (*thyX* CheckF and *thyX* CheckR) span the *thyX* genomic region and so yielded a smaller-sized product upon *thyX* deletion (2427 bp) when compared with wild-type (3159 bp). Colonies 31 and 34 for pASF59 were later shown to be wild-type at the *thyX* locus using the same primers. +, Wild-type H37Rv positive controls; −, distilled H_2_O negative controls. The marker is the upper half of Hyperladder I (Bioline). (c) Schematic diagram showing the region of *thyA* that was deleted and the locations of the *Pst*I sites and the probe used for Southern blotting. (d) Southern blot analysis of three independently generated *M. tuberculosis thyA* deletion strains (1–3) alongside wild-type H37Rv (WT). The *Pst*I site within *thyA* of the wild-type strain is absent within the three deletion strains, and so a larger-sized product was detected (9171 bp compared with 5401 bp).

*thyX* (Rv2754c) was targeted for in-frame deletion within *M. tuberculosis*, as RT-PCR indicated that it is co-transcribed with its downstream genes *dapA* (Rv2753c) and Rv2752c (Fig. 2a; data not shown). After electroporation of pJH-*thyX* into *M. tuberculosis* H37Rv, potential single cross-over recombinants were analysed by PCR (results not shown), to ensure that integration within the strain taken forward had occurred at the correct locus. Upon selection, no double cross-over strains, i.e. *thyX* deletion strains, were obtained under normal conditions, or in the presence of 50, 100 or 500 µg thymidine ml^−1^ (all colonies obtained or 36 colonies were analysed by PCR for each condition; data not shown). This suggests that *thyX* is essential for the survival of *M. tuberculosis*, even in the presence of *thyA* and exogenous thymidine.

To confirm essentiality, an additional copy of *thyX* expressed from its own promoter (on pASF59) was integrated into the *att* site of the *thyX* single cross-over strain, creating a merodiploid strain. Empty vector (pKP203) was also independently integrated into the same single cross-over strain. Upon selection, the wild-type copy of *thyX* was successfully deleted in the presence of pASF59 (11 out of 36 colonies analysed by PCR), but not in the presence of empty vector (0 out of 36 colonies analysed by PCR), confirming that *thyX* is essential under the conditions used (Fig. 2b). The addition of 100 µg thymidine ml^−1^ had no effect on the ability to delete the wild-type copy of *thyX* from either of these strains (data not shown), supporting the hypothesis that *M. tuberculosis* cannot utilize exogenous thymidine because its genome does not encode thymidine kinase (Myllykallio *et al.*, 2002).

### *thyA* (Rv2764c) is not essential

*thyA* (Rv2764c) was targeted for in-frame deletion within *M. tuberculosis*, as RT-PCR indicated that it is co-transcribed with its downstream genes *dfrA* (Rv2763c) and Rv2762c (Fig. 2a; data not shown). We did not assess whether this transcript extends beyond Rv2762c, so it remains possible that *thyA*-Rv2757c forms a single operon. Strains containing the *thyA* deletion were isolated; five of the 28 white, sucrose-resistant, kanamycin-sensitive colonies analysed by PCR gave the products expected for the *thyA* deletion (results not shown), and three of these were further confirmed by Southern blotting (Fig. 2c, d). *M. tuberculosis thyA* deletion strain 1 from Fig. 2(d), designated Δ*thyA*, was used for all further experiments.

### *thyA* deletion confers an *in vitro* growth defect

**Fig. 3.**
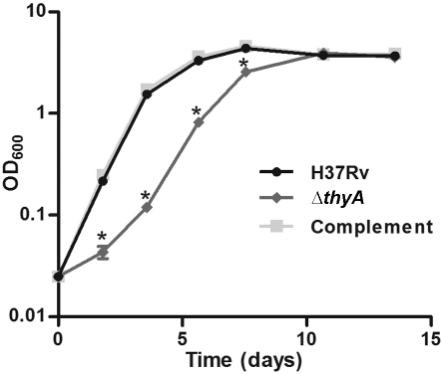
*In vitro* growth of *M. tuberculosis* Δ*thyA*. *In vitro* growth curves of *M. tuberculosis* H37Rv, Δ*thyA* and the complemented strain (Δ*thyA* containing pASF63). Strains were grown in modified Dubos medium containing 10 % albumin and 0.2 % (w/v) glycerol. The data are means of three biological replicates; error bars, sd. The wild-type H37Rv and complemented strain curves overlap. Times when the OD_600_ of *M. tuberculosis* Δ*thyA* was significantly lower than that of wild-type H37Rv and the complemented strain are indicated (*; Student’s *t* test, *P*<0.01).

An immediate growth defect was seen when attempting to grow *M. tuberculosis* Δ*thyA* with rolling at 2 r.p.m. in modified Dubos medium [containing 4 % albumin and 0.2 % (w/v) glycerol]. The strain grew noticeably more slowly than wild-type H37Rv and began to clump at OD_600_ ~0.5. Upon increasing the albumin concentration within the medium to 10 %, Δ*thyA* no longer clumped and so it was possible to produce growth curves, alongside H37Rv and the complemented strain (Δ*thyA* containing pASF63). The OD_600_ of *M. tuberculosis* Δ*thyA* differed significantly from that of wild-type H37Rv at four of the seven time points (Fig. 3). *M. tuberculosis* Δ*thyA* appears to have a prolonged lag phase compared with wild-type H37Rv, and this growth defect was restored to the wild-type phenotype in the complemented strain (Fig. 3). The addition of thymidine (100 µg ml^−1^) did not have any effect on the growth of these three strains (results not shown), again supporting the hypothesis that *M. tuberculosis* cannot utilize exogenous thymidine. From this point onward, all liquid media contained 10 % albumin.

### *thyA* deletion does not affect *in vivo* growth

**Fig. 4.**
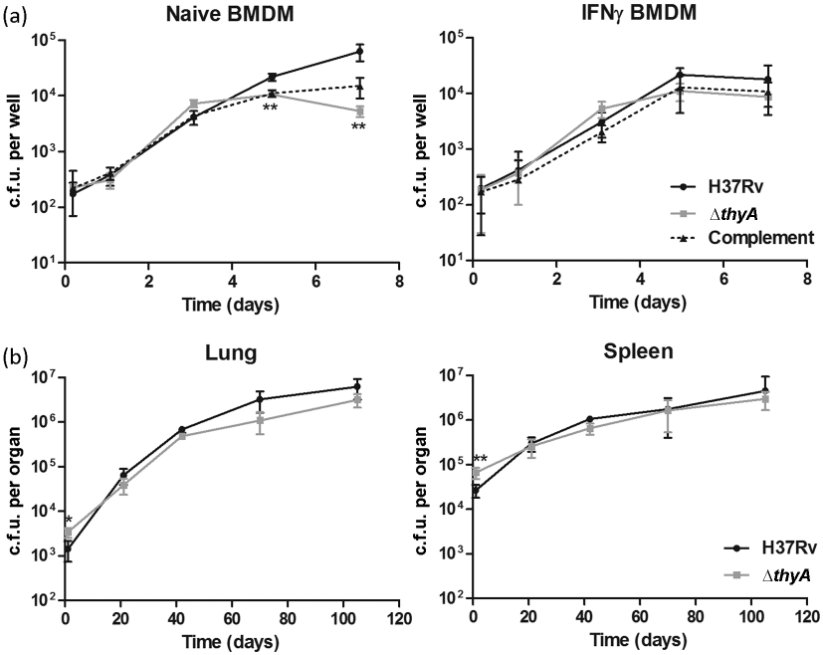
*In vivo* growth of *M. tuberculosis* Δ*thyA.* Growth of *M. tuberculosis* H37Rv and Δ*thyA* was assessed within (a) murine BMDM (alongside the complemented strain; Δ*thyA* containing pASF63) and (b) BALB/c mice. The data are means of three macrophage infections or the lungs and spleens of four mice; error bars, sd. Times when the quantity of *M. tuberculosis* Δ*thyA* was significantly different from that of wild-type H37Rv are indicated (* or **; Student’s *t* test, *P*<0.05 or *P*<0.01, respectively).

Infection models were utilized to assess the effect of *thyA* deletion on *in vivo* growth. *M. tuberculosis* H37Rv and Δ*thyA* were used to infect murine BMDM (at an m.o.i. of 0.1 : 1 for 4 h) and immunocompetent BALB/c mice (intravenously). Bacteria within macrophages and within the lungs and spleens of mice were enumerated at various time points post-infection to assess the survival and multiplication of each strain. Although the quantity of *M. tuberculosis* Δ*thyA* within naive macrophages differed significantly from equivalent wild-type H37Rv numbers at later time points (Fig. 4a), this was not the case in the activated macrophage and mouse model analysis (Fig. 4a, b). Therefore, we concluded that the deletion of *thyA* does not affect *M. tuberculosis in vivo* growth.

### *thyX* expression is not elevated to compensate for the loss of *thyA*

**Fig. 5.**
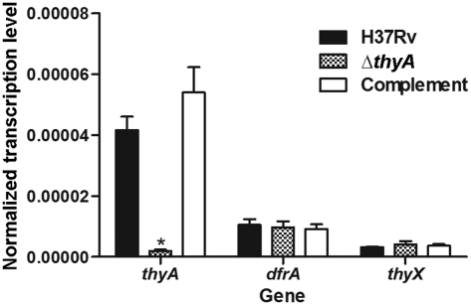
*thyA*, *dfrA* and *thyX* expression levels in *M. tuberculosis* Δ*thyA*. qRT-PCR was performed on RNA extracts of *M. tuberculosis* H37Rv, Δ*thyA* and the complemented strain (Δ*thyA* containing pASF63), grown to exponential phase (OD_600_ 0.8). The data are means of at least three biological replicates (each assayed in triplicate), normalized to corresponding *rrs* (16S rRNA) values; error bars, sd. Only the expression level of *thyA* was significantly lower within *M. tuberculosis* Δ*thyA* compared with equivalent wild-type H37Rv and complemented strain levels (*; Student’s *t* test, *P*<0.01).

As our studies had shown that the expression of *thyX* was much lower than that of *thyA* under all conditions tested, yet it was possible to delete *thyA* we wondered whether the expression of *thyX* might be elevated in the *thyA* deletion strain as a means of compensating for its absence. To assess this, the relative expression levels of *thyA*, *dfrA* and *thyX* within *M. tuberculosis* H37Rv, Δ*thyA* and the complemented strain were quantified by qRT-PCR (Fig. 5). The results confirmed that there was significantly less *thyA* expression within *M. tuberculosis* Δ*thyA* when compared with wild-type H37Rv levels, and that *thyA* expression was restored to wild-type levels within the complemented strain. Furthermore, *dfrA* expression was unaffected by *thyA* deletion, so the deletion does not appear to have resulted in downstream polar effects. Importantly, the expression of *thyX* was also unchanged in the *thyA* deletion strain. This suggests that although both *M. tuberculosis* ThyA and ThyX proteins are known to have TS activity *in vitro* (Hunter *et al.*, 2008), *thyX* expression is not upregulated to compensate for the lack of *thyA* expression.

### *thyA* deletion confers PAS resistance

**Fig. 6.**
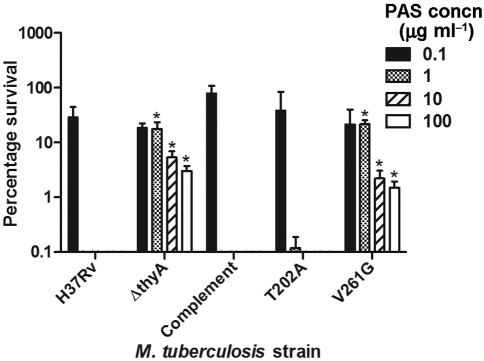
PAS susceptibility of *M. tuberculosis* Δ*thyA* and the effects of complementing with mutated versions of *thyA.* PAS susceptibility of *M. tuberculosis* H37Rv, Δ*thyA* and three complementing strains, presented as percentage survival relative to untreated samples. The complemented strain (Δ*thyA* containing pASF63) expressed wild-type *thyA*, while T202A and V261G (Δ*thyA* containing pASF70 and pASF71, respectively) expressed the indicated mutated versions of *thyA*. The data are means of three biological replicates; error bars, sd. An asterisk indicates that the percentage survival of a particular strain was significantly greater than that of the wild-type H37Rv (*; Student’s *t* test, *P*<0.02).

As some clinical *M. tuberculosis* PAS-resistant strains have mutations within *thyA* (Leung *et al.*, 2010; Mathys *et al.*, 2009; Rengarajan *et al.*, 2004; Zhang *et al.*, 2007), we investigated whether *thyA* deletion confers PAS resistance. Briefly, cultures were grown to exponential phase and plated onto agar containing increasing concentrations of PAS (up to 100 µg ml^−1^). The percentage survival of each strain for each PAS concentration was then calculated compared with that of cells plated without any PAS. The survival of *M. tuberculosis* Δ*thyA* was significantly greater than that of wild-type H37Rv at all concentrations ≥1 µg PAS ml^−1^, and this phenotype was restored to wild-type in the complemented strain (Fig. 6). Therefore, *thyA* deletion confers PAS resistance.

### T202A and V261G mutations, associated with clinical PAS resistance, render ThyA functional and non-functional, respectively

To further investigate the relationship between ThyA and PAS activity, we analysed the effect of complementing *M. tuberculosis* Δ*thyA* with *thyA* containing mutations associated with PAS resistance. The *thyA* T202V (ACC→GCC) and V261G (GTC→GGC) mutations have been identified within some clinical *M. tuberculosis* PAS-resistant strains, and are thought to affect the structural stability and active catalytic site of the enzyme, respectively (Leung *et al.*, 2010; Mathys *et al.*, 2009; Rengarajan *et al.*, 2004; Zhang *et al.*, 2007). These mutations were independently introduced into the *thyA* complementation plasmid pASF63 by SDM, yielding pASF70 and pASF71, respectively. *M. tuberculosis* Δ*thyA* complemented with *thyA* T202A (pASF70) was able to restore the *in vitro* growth defect of the deletion strain back to the wild-type phenotype, while the strain complemented with *thyA* V261G (pASF71) retained the growth defect of the deletion strain (data not shown). In addition, *thyA* T202A was able to complement the PAS resistance seen for the deletion strain, while *thyA* V261G could not (Fig. 6). qRT-PCR analysis showed that the introduction of these mutations had no effect on the *thyA* transcription level from the complementation plasmid (data not shown). Therefore, ThyA T202A appears to be functional and ThyA V261G appears to be non-functional. It seems that functional ThyA is required for *M. tuberculosis* to be sensitive to PAS.

## Discussion

To gain insight into the biological roles of *M. tuberculosis thyA* and *thyX*, we first analysed the *in vitro* and *in vivo* expression levels of these two genes by qRT-PCR. Contrary to earlier microarray analysis (Talaat *et al.*, 2004), expression of both *thyA* and *thyX* was detected. Under all conditions tested, *thyA* expression exceeded that of *thyX*. We were unable to identify a condition where *thyX* expression alone was increased. However, expression of both genes significantly increased upon *in vitro* acid exposure (pH 5.4) and when grown within naive and activated murine macrophages, so both ThyA and ThyX may play an important role *in vivo*. To our knowledge only one other study has shown that *thyA* is upregulated *in vivo* (within THP-1 human macrophages; Fontán *et al.*, 2008), while Schnappinger *et al.* (2003) showed that *thyA* and *thyX* expression was unchanged in murine macrophages. Again, this illustrates that interesting observations can sometimes be missed when using microarray technology. Microarrays suffer from inherent relatively large variability in expression levels for genes expressed at low levels, meaning that the expression changes of these genes are not shown to be significantly different (Butte, 2002; Chuchana *et al.*, 2007).

In agreement with transposon site hybridization (TraSH) experiments (Sassetti *et al.*, 2003), we showed that *M. tuberculosis thyX* is essential on solid media *in vitro*. The requirement for *thyX* in the presence of *thyA* implies that the essential function of ThyX is something other than dTMP synthase. Earlier studies have indeed questioned whether the true biological substrates of ThyX have been identified, as many of these enzymes (including ThyX from *M. tuberculosis* and *C.* *glutamicum*) have very low TS catalytic activity (Agrawal *et al.*, 2004; Hunter *et al.*, 2008; Kan *et al.*, 2010). It should be noted that *thyX* from *C.* *glutamicum* is only thought to be essential upon reaching stationary phase (Park *et al.*, 2010), and so the biological roles of ThyX appear to differ between these two relatively closely related species. Importantly, we have shown that this enzyme is a plausible tuberculosis drug target, and our collaborators have recently published their findings that compounds specifically designed to inhibit *M. tuberculosis* ThyX are effective, with no activity against ThyA (Kögler *et al.*, 2011). It would be of great interest to identify the primary essential function of ThyX in *M. tuberculosis*, and this could possibly be achieved using a metabolomics approach (de Carvalho *et al.*, 2010).

We successfully deleted *thyA* from the *M. tuberculosis* genome, and this deletion was shown to confer an *in vitro* growth defect that was not observed *in vivo*. *M. tuberculosis* has evolved to grow within its human host, and culture medium is a very unnatural environment for the bacterium. The murine macrophage and animal models used within this study are much more representative of the natural *M. tuberculosis* environment. The *in vitro* experiments do support the hypothesis that *M. tuberculosis* cannot utilize exogenous thymidine because its genome does not encode thymidine kinase (Myllykallio *et al.*, 2002). It therefore appears as though ThyX performs TS activity within *M. tuberculosis* Δ*thyA* at a rate sufficient for normal growth *in vivo*, but that the rate *in vitro* is less than optimal. This could be due to a change in the *thyX* transcription rate and/or ThyX TS activity. We have already shown that *M. tuberculosis thyX* mRNA levels are higher *in vivo* than *in vitro*. Also, *Campylobacter jejuni* ThyX is known to be inhibited by oxygen (Giladi *et al.*, 2002), so *M. tuberculosis* ThyX may be more active *in vivo* under conditions of limited oxygen availability than *in vitro*. The *in vitro* expression level of *thyX* is unchanged within *M. tuberculosis* Δ*thyA*, indicating that the transcriptional regulation of the two TS genes is probably not linked. It would be interesting to know whether *thyX* is actually essential for *in vivo* growth. This could be investigated by switching (Pashley & Parish, 2003) the existing complementation plasmid within a strain where wild-type *thyX* was successfully deleted, with a plasmid expressing *thyX* from a regulated promoter. The strain could then be grown *in vitro* with *thyX* being expressed and subsequently used to infect macrophages or mice without *thyX* expression (Boldrin *et al.*, 2010; Carroll *et al.*, 2005; Guo *et al.*, 2007). Unfortunately, these experiments are beyond the scope of this study.

Some clinical *M. tuberculosis* PAS-resistant strains contain point mutations within *thyA* (Leung *et al.*, 2010; Mathys *et al.*, 2009; Rengarajan *et al.*, 2004; Zhang *et al.*, 2007). We have conclusively demonstrated that *thyA* deletion confers *M. tuberculosis* PAS resistance. We also showed that ThyA T202A appears to be functional and ThyA V261G appears to be non-functional. This finding is supported by a recent study which demonstrated that the *thyA* T202A mutation is a marker for the Latin American Mediterranean lineage of *M. tuberculosis* and is not associated with PAS resistance (Feuerriegel *et al.*, 2010). The mode of action of PAS is currently unknown. PAS has structural similarities to sulphonamides, which act as competitive inhibitors of dihydropteroate synthase (FolP1 and FolP2), and dihydropteroate is a precursor of DHF. However, PAS appears to be a poor inhibitor of *M. tuberculosis* FolP1 *in vitro* (Nopponpunth *et al.*, 1999), and mutations within *M. tuberculosis folP1* and *folP2* have not been found to be associated with PAS resistance (Mathys *et al.*, 2009). It has been suggested that PAS may be a pro-drug whose activation requires viable ThyA (Mathys *et al.*, 2009; Rengarajan *et al.*, 2004), but only approximately 36 % of clinical and spontaneous *M. tuberculosis* PAS-resistant strains have mutations in *thyA* (Mathys *et al.*, 2009; Zhang *et al.*, 2007). Also, *Mycobacterium bovis* BCG *thyA* mutations confer resistance to 8710, a trimethoprim analogue that antagonizes mycobacterial DHFR, in addition to PAS (Rengarajan *et al.*, 2004). Therefore, PAS appears to target an unknown component of folate biosynthesis, and as ThyA is a major consumer of THF, strains mutated in *thyA* are able to withstand the pressure on the folate cycle (Leduc *et al.*, 2007; Rengarajan *et al.*, 2004). Nevertheless, *thyA* sequencing has proved effective at predicting PAS treatment outcome (Leung *et al.*, 2010).

## References

[r1] Agrawal N. Lesley S. A. Kuhn P. Kohen A. **(**2004**).** Mechanistic studies of a flavin-dependent thymidylate synthase. Biochemistry 43, 10295–10301. 10.1021/bi0490439 15301527

[r2] Ahmed N. Hasnain S. E. **(**2004**).** Genomics of *Mycobacterium tuberculosis*: old threats & new trends. Indian J Med Res 120, 207–212. 15520478

[r3] Boldrin F. Casonato S. Dainese E. Sala C. Dhar N. Palù G. Riccardi G. Cole S. T. Manganelli R. **(**2010**).** Development of a repressible mycobacterial promoter system based on two transcriptional repressors. Nucleic Acids Res 38, e134. 10.1093/nar/gkq235 20406773 PMC2896539

[r4] Butcher P. D. Mangan J. Monahan I. M. **(**1998**).** Intracellular gene expression: analysis of RNA from mycobacteria in macrophages using RT-PCR. In Methods in Molecular Biology, Mycobacteria Protocols, 101, pp. 285–306. Edited by Parish T. Stoker N. G. Totowa, NJ: Humana Press 10.1385/0-89603-471-2:285 9921487

[r5] Butte A. **(**2002**).** The use and analysis of microarray data. Nat Rev Drug Discov 1, 951–960. 10.1038/nrd961 12461517

[r6] Carreras C. W. Santi D. V. **(**1995**).** The catalytic mechanism and structure of thymidylate synthase. Annu Rev Biochem 64, 721–762. 10.1146/annurev.bi.64.070195.003445 7574499

[r7] Carroll P. Muttucumaru D. G. Parish T. **(**2005**).** Use of a tetracycline-inducible system for conditional expression in *Mycobacterium tuberculosis* and *Mycobacterium smegmatis*. Appl Environ Microbiol 71, 3077–3084. 10.1128/AEM.71.6.3077-3084.2005 15933004 PMC1151860

[r8] Chuchana P. Marchand D. Nugoli M. Rodriguez C. Molinari N. Garcia-Sanz J. A. **(**2007**).** An adaptation of the LMS method to determine expression variations in profiling data. Nucleic Acids Res 35, e71. 10.1093/nar/gkm093 17459890 PMC1888829

[r9] de Carvalho L. P. S. Zhao H. Dickinson C. E. Arango N. M. Lima C. D. Fischer S. M. Ouerfelli O. Nathan C. Rhee K. Y. **(**2010**).** Activity-based metabolomic profiling of enzymatic function: identification of Rv1248c as a mycobacterial 2-hydroxy-3-oxoadipate synthase. Chem Biol 17, 323–332. 10.1016/j.chembiol.2010.03.009 20416504 PMC2878197

[r10] Feuerriegel S. Köser C. Trübe L. Archer J. Rüsch Gerdes S. Richter E. Niemann S. **(**2010**).** Thr202Ala in *thyA* is a marker for the Latin American Mediterranean lineage of the *Mycobacterium tuberculosis* complex rather than *para*-aminosalicylic acid resistance. Antimicrob Agents Chemother 54, 4794–4798. 10.1128/AAC.00738-10 20805400 PMC2976163

[r11] Fivian-Hughes A. S. Davis E. O. **(**2010**).** Analyzing the regulatory role of the HigA antitoxin within *Mycobacterium tuberculosis*. J Bacteriol 192, 4348–4356. 10.1128/JB.00454-10 20585061 PMC2937366

[r12] Fontán P. Aris V. Ghanny S. Soteropoulos P. Smith I. **(**2008**).** Global transcriptional profile of *Mycobacterium tuberculosis* during THP-1 human macrophage infection. Infect Immun 76, 717–725. 10.1128/IAI.00974-07 18070897 PMC2223452

[r13] Gattis S. G. Palfey B. A. **(**2005**).** Direct observation of the participation of flavin in product formation by *thyX*-encoded thymidylate synthase. J Am Chem Soc 127, 832–833. 10.1021/ja0432214 15656610

[r14] Giladi M. Bitan-Banin G. Mevarech M. Ortenberg R. **(**2002**).** Genetic evidence for a novel thymidylate synthase in the halophilic archaeon *Halobacterium salinarum* and in *Campylobacter jejuni*. FEMS Microbiol Lett 216, 105–109. 10.1111/j.1574-6968.2002.tb11422.x 12423760

[r15] Gopaul K. K. **(**2002**).** *Transcription of the Mycobacterium tuberculosis recA gene*. PhD thesis, University College London.

[r16] Guo X. V. Monteleone M. Klotzsche M. Kamionka A. Hillen W. Braunstein M. Ehrt S. Schnappinger D. **(**2007**).** Silencing *Mycobacterium smegmatis* by using tetracycline repressors. J Bacteriol 189, 4614–4623. 10.1128/JB.00216-07 17483222 PMC1913471

[r17] Haydel S. E. **(**2010**).** Extensively drug-resistant tuberculosis: A sign of the times and an impetus for antimicrobial discovery. Pharmaceuticals (Basel) 3, 2268–2290. 21170297 10.3390/ph3072268 PMC3002907

[r18] Hunter J. H. Gujjar R. Pang C. K. Rathod P. K. **(**2008**).** Kinetics and ligand-binding preferences of *Mycobacterium tuberculosis* thymidylate synthases, ThyA and ThyX. PLoS ONE 3, e2237. 10.1371/journal.pone.0002237 18493582 PMC2386288

[r19] Kan S. C. Liu J. S. Hu H. Y. Chang C. M. Lin W. D. Wang W. C. Hsu W. H. **(**2010**).** Biochemical characterization of two thymidylate synthases in *Corynebacterium glutamicum* NCHU 87078. Biochim Biophys Acta 1804, 1751–1759. 20595007 10.1016/j.bbapap.2010.05.006

[r20] Koehn E. M. Fleischmann T. Conrad J. A. Palfey B. A. Lesley S. A. Mathews I. I. Kohen A. **(**2009**).** An unusual mechanism of thymidylate biosynthesis in organisms containing the *thyX* gene. Nature 458, 919–923. 10.1038/nature07973 19370033 PMC2759699

[r21] Kögler M. Vanderhoydonck B. De Jonghe S. Rozenski J. Van Belle K. Herman J. Louat T. Parchina A. Sibley C. **& other authors (**2011**).** Synthesis and evaluation of 5-substituted 2′-deoxyuridine monophosphate analogues as inhibitors of flavin-dependent thymidylate synthase in *Mycobacterium tuberculosis*. J Med Chem 54, 4847–4862. 10.1021/jm2004688 21657202

[r22] Koul A. Arnoult E. Lounis N. Guillemont J. Andries K. **(**2011**).** The challenge of new drug discovery for tuberculosis. Nature 469, 483–490. 10.1038/nature09657 21270886

[r23] Kuhn P. Lesley S. A. Mathews I. I. Canaves J. M. Brinen L. S. Dai X. Deacon A. M. Elsliger M. A. Eshaghi S. **& other authors (**2002**).** Crystal structure of Thy1, a thymidylate synthase complementing protein from *Thermotoga maritima* at 2.25 Å resolution. Proteins 49, 142–145. 10.1002/prot.10202 12211025

[r24] Leduc D. Escartin F. Nijhout H. F. Reed M. C. Liebl U. Skouloubris S. Myllykallio H. **(**2007**).** Flavin-dependent thymidylate synthase ThyX activity: implications for the folate cycle in bacteria. J Bacteriol 189, 8537–8545. 10.1128/JB.01380-07 17890305 PMC2168944

[r25] Leung K. L. Yip C. W. Yeung Y. L. Wong K. L. Chan W. Y. Chan M. Y. Kam K. M. **(**2010**).** Usefulness of resistant gene markers for predicting treatment outcome on second-line anti-tuberculosis drugs. J Appl Microbiol 109, 2087–2094. 10.1111/j.1365-2672.2010.04840.x 20854453

[r26] Mathys V. Wintjens R. Lefevre P. Bertout J. Singhal A. Kiass M. Kurepina N. Wang X. M. Mathema B. **& other authors (**2009**).** Molecular genetics of *para*-aminosalicylic acid resistance in clinical isolates and spontaneous mutants of *Mycobacterium tuberculosis*. Antimicrob Agents Chemother 53, 2100–2109. 10.1128/AAC.01197-08 19237648 PMC2681553

[r27] Myllykallio H. Lipowski G. Leduc D. Filee J. Forterre P. Liebl U. **(**2002**).** An alternative flavin-dependent mechanism for thymidylate synthesis. Science 297, 105–107. 10.1126/science.1072113 12029065

[r28] Myllykallio H. Leduc D. Filee J. Liebl U. **(**2003**).** Life without dihydrofolate reductase FolA. Trends Microbiol 11, 220–223. 10.1016/S0966-842X(03)00101-X 12781525

[r29] Nopponpunth V. Sirawaraporn W. Greene P. J. Santi D. V. **(**1999**).** Cloning and expression of *Mycobacterium tuberculosis* and *Mycobacterium leprae* dihydropteroate synthase in *Escherichia coli*. J Bacteriol 181, 6814–6821. 10542185 10.1128/jb.181.21.6814-6821.1999 PMC94148

[r30] Parish T. Stoker N. G. **(**2000**).** Use of a flexible cassette method to generate a double unmarked *Mycobacterium tuberculosis tlyA plcABC* mutant by gene replacement. Microbiology 146, 1969–1975. 10931901 10.1099/00221287-146-8-1969

[r31] Park M. Cho S. Lee H. Sibley C. H. Rhie H. **(**2010**).** Alternative thymidylate synthase, ThyX, involved in *Corynebacterium glutamicum* ATCC 13032 survival during stationary growth phase. FEMS Microbiol Lett 307, 128–134. 10.1111/j.1574-6968.2010.01971.x 20636973

[r32] Pashley C. A. Parish T. **(**2003**).** Efficient switching of mycobacteriophage L5-based integrating plasmids in *Mycobacterium tuberculosis*. FEMS Microbiol Lett 229, 211–215. 10.1016/S0378-1097(03)00823-1 14680701

[r33] Rengarajan J. Sassetti C. M. Naroditskaya V. Sloutsky A. Bloom B. R. Rubin E. J. **(**2004**).** The folate pathway is a target for resistance to the drug *para*-aminosalicylic acid (PAS) in mycobacteria. Mol Microbiol 53, 275–282. 10.1111/j.1365-2958.2004.04120.x 15225321

[r34] Rickman L. Scott C. Hunt D. M. Hutchinson T. Menéndez M. C. Whalan R. Hinds J. Colston M. J. Green J. Buxton R. S. **(**2005**).** A member of the cAMP receptor protein family of transcription regulators in *Mycobacterium tuberculosis* is required for virulence in mice and controls transcription of the *rpfA* gene coding for a resuscitation promoting factor. Mol Microbiol 56, 1274–1286. 10.1111/j.1365-2958.2005.04609.x 15882420 PMC2964915

[r34a] Rossi F. Khanduja J. S. Bartoluzzi A. Houghton J. Sander P. Güthlein C. Davis E. O. Springer B. Böttger E. C. **& other authors (**2011**).** The biological and structural characterization of *Mycobacterium tuberculosis* UvrA provides novel insights into its mechanism of action. Nucleic Acids Res 39, 7316–7328. 10.1111/j.1365-2958.2005.04609.x 21622956 PMC3167621

[r35] Sampathkumar P. Turley S. Ulmer J. E. Rhie H. G. Sibley C. H. Hol W. G. J. **(**2005**).** Structure of the *Mycobacterium tuberculosis* flavin dependent thymidylate synthase (MtbThyX) at 2.0 Å resolution. J Mol Biol 352, 1091–1104. 10.1016/j.jmb.2005.07.071 16139296

[r36] Sampathkumar P. Turley S. Sibley C. H. Hol W. G. J. **(**2006**).** NADP^+^ expels both the co-factor and a substrate analog from the *Mycobacterium tuberculosis* ThyX active site: opportunities for anti-bacterial drug design. J Mol Biol 360, 1–6. 10.1016/j.jmb.2006.04.061 16730023

[r37] Sassetti C. M. Boyd D. H. Rubin E. J. **(**2003**).** Genes required for mycobacterial growth defined by high density mutagenesis. Mol Microbiol 48, 77–84. 10.1046/j.1365-2958.2003.03425.x 12657046

[r38] Schnappinger D. Ehrt S. Voskuil M. I. Liu Y. Mangan J. A. Monahan I. M. Dolganov G. Efron B. Butcher P. D. **& other authors (**2003**).** Transcriptional adaptation of *Mycobacterium tuberculosis* within macrophages: insights into the phagosomal environment. J Exp Med 198, 693–704. 10.1084/jem.20030846 12953091 PMC2194186

[r39] Spivey V. L. Molle V. Whalan R. H. Rodgers A. Leiba J. Stach L. Walker K. B. Smerdon S. J. Buxton R. S. **(**2011**).** Forkhead-associated (FHA) domain containing ABC transporter Rv1747 is positively regulated by Ser/Thr phosphorylation in *Mycobacterium tuberculosis*. J Biol Chem 286, 26198–26209. 10.1074/jbc.M111.246132 21622570 PMC3138270

[r40] Springer B. Sander P. Sedlacek L. Ellrott K. Böttger E. C. **(**2001**).** Instability and site-specific excision of integration-proficient mycobacteriophage L5 plasmids: development of stably maintained integrative vectors. Int J Med Microbiol 290, 669–675. 10.1016/S1438-4221(01)80004-7 11310445

[r41] Talaat A. M. Lyons R. Howard S. T. Johnston S. A. **(**2004**).** The temporal expression profile of *Mycobacterium tuberculosis* infection in mice. Proc Natl Acad Sci U S A 101, 4602–4607. 10.1073/pnas.0306023101 15070764 PMC384793

[r42] Ulmer J. E. Boum Y. Thouvenel C. D. Myllykallio H. Sibley C. H. **(**2008**).** Functional analysis of the *Mycobacterium tuberculosis* FAD-dependent thymidylate synthase, ThyX, reveals new amino acid residues contributing to an extended ThyX motif. J Bacteriol 190, 2056–2064. 10.1128/JB.01094-07 18192395 PMC2258874

[r43] WHO **(**2009**).** Global tuberculosis control: epidemiology, strategy, financing. Geneva, Switzerland: WHO.

[r44] Zhang Z. D. Zhao Y. L. Li Z. H. Jia H. Y. Liu Y. H. Chen X. Liu Z. Q. Du B. P. Xing A. Y. Ma Y. **(**2007**).** [Mutations in the thymidylate synthase gene is a major mechanism in the *para*-aminosalicylic acid resistance of *M. tuberculosis*]. Zhonghua Jie He He Hu Xi Za Zhi 30, 683–685 (in Chinese). 18070553

[r45] Zignol M. Hosseini M. S. Wright A. Weezenbeek C. L. Nunn P. Watt C. J. Williams B. G. Dye C. **(**2006**).** Global incidence of multidrug-resistant tuberculosis. J Infect Dis 194, 479–485. 10.1086/505877 16845631

